# Annotations

**Published:** 1895-04-06

**Authors:** 


### ANNOTATIONS.

### Cheap Dispensing.

The public as a whole loves value for its money, and will certainly in practice "buy in the cheapest market and sell in the dearest." This is not to be blamed. In carrying out the maxim, however, it is of prime importance to any particular buyer that he should know which is the cheapest market. Physic is an article of increasing consumption. People need it more than they did, and they know its uses better. The sale and purchase of physic will more and more tend to be influenced by the ordinary maxims of the market. But with physic mere quantity and mere price, so important in most other things, are of quite secondary importance. The really important thing is to have exactly what is required. The doctor who prescribes drugs nowadays does not prescribe at random, or mix up half-a-dozen more or less serviceable substances in the hope that if one does not answer the purpose another will. On the contrary, he knows with great certainty the properties of the drugs he uses, and with a precision which is almost absolute their physiological effects upon the human organism. When he prescribes, therefore, he prescribes particular substances in such precise doses as to produce exactly the physiological effects he desires to bring about. Is it not plain, then, that every person who takes physic should assure himself positively of the quality, whatever he does about the price? The price is secondary, absolutely of no importance compared with the quality. Besides, how small an item mere physic is, after all, in the whole year's expenditure! Even in a large household, the difference between the best drugs, paid for at the best prices, and the worst, paid for at the lowest prices, cannot amount to more than two or three pounds a year; in many houses a few shillings would be nearer the sum. If a physician may presume to give advice to the public, he would say, "Economise as much as you please on things not necessary to life and health, but in dealing with things like food and physic, always make sure of having the best, whatever the cost."

### The Annual Meeting.

For some time past it has been our endeavour to render some assistance to hospitals and charitable institutions by sending a representative to their annual meetings, as these come round, with a view to the publication of a more or less detailed report of the proceedings in The Hospital. In so doing it has been increasingly borne in upon us that the most marked feature of these meetings is their uniformity of dulness. The speakers, for the most part, say the same old thing in the same old way (though there are, of course, bright exceptions). The proceedings are too often gone through in a perfunctory manner, in a spirit which makes its indifference felt throughout and beyond the meeting. Thus, instead of those present being aroused to a lively and lasting interest, and stimulated to further offers of help in money or personal service, they go away with a sense of dissatisfaction at having devoted valuable time to a dreary duty which does no good, and which they hope need not be repeated another year. This is not as it should be, and it cannot be too strongly impressed upon hospitals and charities generally that these same annual gatherings are splendid opportunities, too often lost, when enthusiasm might and ought to be aroused, subscriptions and donations augmented, and valuable personal help and influence gained. More heart must be put into these occasions by the selection of a chairman who is awake and full of life and love for good works. The speeches of those who preside at and attend annual meetings can only be adequately alive if the hearts of the speakers have been with the work all the year through. A striking proof of what can be done by individual effort was shown at the meeting of the Poplar Hospital for Accidents which took place last week, where no one present who could read between the lines could have failed to be impressed by the spirit of genuine and eager co-operation manifested. Mr. Sydney Holland in encouraging the attendance of the working men for whose benefit the hospital exists, managed to cement feelings of good fellowship, and to draw together into a harmonious whole all the many interests of which a hospital is the centre. As a result nearly £50,000 has been raised at Poplar for a merely nominal cost, the hospital is free from debt, and probably more efficiently worked than any similar institution. We do most earnestly recommend to the notice of those who are responsible for organising annual meetings the importance of a new departure in the directions indicated. More heart, energy, and life in the proceedings of these meetings must yield abundant and good fruit, helpful to both the institutions and the public.

### Mushrooms and Manure-heaps.

On several occasions lately it has happened to the writer to meet with cases of vomiting, diarrhœa, colicky pains, and great prostration, lasting twenty-four to forty-eight hours, as the result of eating artificially-grown mushrooms. As grown in old grass pastures, mushrooms are agreeable and excellent eating, especially if cooked properly and cooked fresh. Even as produced artificially for the market, they are often quite wholesome if washed clean and cooked early. But, as is well known, mushrooms belong to an order of vegetables of a somewhat low organisation, and they grow and reproduce themselves with remarkable rapidity when sown in decomposing vegetable matter. Many growers take advantage of this fact to cultivate mushrooms on manure-heaps—heaps, that is to say, not of ordinary farmyard manure, but of the vile and rotting filth of every description which is gathered together in large towns and delivered to suburban and country mushroom-growers by horse-waggon or train. Now, plants take up into themselves the very stuff, modified, on which they grow. Mushrooms grown on matter of this sort select from it those parts which they are able to assimilate. But the arrangement of the "cap" of the mushroom enables it also to absorb the vapour of the manure, which is a dangerous poison to man and other animals. Thus, not only do mushrooms grown on such manure-heaps as we have described assimilate deadly poison, but the scores or hundreds of radiating plates of which they principally consist are in practice little better than traps for the catching and retaining of more deadly poisons still. The moral is, never grow mushrooms on manure-heaps, especially on heaps of town manure, but only on beds specially prepared with a view to health. The wholesomest of all mushrooms are those grown among the fairies in old pastures.

